# The Dyspnea-ALS-Scale (DALS-15) optimizes individual treatment in patients with amyotrophic lateral sclerosis (ALS) suffering from dyspnea

**DOI:** 10.1186/s12955-019-1167-0

**Published:** 2019-06-03

**Authors:** Susanne Vogt, Stefanie Schreiber, Hans-Jochen Heinze, Reinhard Dengler, Susanne Petri, Stefan Vielhaber

**Affiliations:** 1 Department of Neurology, Otto-von-Guericke University, Leipziger Str. 44, D-39120 Magdeburg, Germany; 2 Department of Neurology, Hannover Medical School, Carl-Neuberg-Str. 1, D-30625 Hannover, Germany; 3 German Center for Neurodegenerative Diseases, Leipziger Str. 44, D-39120 Magdeburg, Germany; 4 Leibniz Institute for Neurobiology, Brenneckestraße 6, D-39118 Magdeburg, Germany

**Keywords:** Amyotrophic lateral sclerosis, Motor neuron disease, Dyspnea assessment, Patient-reported outcome measure, Pulmonary function tests, Non-invasive ventilation, Symptom management

## Abstract

**Background:**

Dyspnea is frequent in amyotrophic lateral sclerosis (ALS) and one of the most bothersome symptoms. The recently developed Dyspnea-ALS-Scale (DALS-15) is a disease-specific patient-reported outcome to detect and quantify dyspnea.

**Objectives:**

To analyze in a case-based approach the diagnostic and clinical implications and the benefit of the DALS-15 for individual patients in daily clinical routine.

**Methods:**

Dyspnea was assessed by the 15-item comprising DALS-15 in two patients with ALS. Spirometry was performed and blood gases were analyzed. Results were evaluated in the clinical context of the respective patients.

**Results:**

In one patient the presence of dyspnea detected by the DALS-15 indicated noninvasive ventilation (NIV) although forced vital capacity (FVC) and blood gas analysis were well preserved. After NIV implementation, the DALS-15 was helpful to determine the patient’s need for medication, the timing of NIV titration and the adaptation of NIV sessions. In another patient, who was anarthric and no longer able to perform spirometry due to severe bulbar impairment, the DALS-15 allowed a standardized assessment of dyspnea-related distress independently of bulbar dysfunction.

**Conclusion:**

The DALS-15 provides a deeper insight into the respiratory status of individual patients. It helps to diagnose respiratory impairment in patients in whom NIV should be considered although FVC and blood gas results do not reveal indication for NIV. It is also valuable for the guidance of patients in later stages of respiratory impairment when NIV is already implemented, and in patients with severe bulbar dysfunction. The DALS-15 can improve specific symptom management and coordination of care and therefore has the potential to optimize individual treatment in ALS patients with dyspnea.

## Background

Amyotrophic lateral sclerosis (ALS) is a devastating neurodegenerative disease with rapidly progressive loss of motor function. Most patients die within 3 to 5 years of symptom development due to respiratory complications and ventilatory failure [1]*.* The main focus of current clinical care in ALS is centered on alleviating symptoms. One of the most bothersome symptoms is dyspnea [2–5] occurring in about 80% of the patients during the course of the disease [6].

A recently published algorithm on decision making regarding initiation of noninvasive ventilation (NIV) in patients with ALS explicitly mentions the symptom of dyspnea as the first criterion to consider NIV [7]. Hence, regular evaluation of dyspnea during the course of the disease is a critical part of patient care in ALS. However, as the absence of dyspnea does not mean that there is no respiratory impairment, the algorithm requires successive spirometry (forced vital capacity, FVC < 50% or probable 75%) and polygraphy as quantifiable and objective parameters to initiate NIV [7]. Up to now, NIV is the strongest evidence-based therapy to prolong survival and improve quality of life in ALS [8]. Hence, the assessment of dyspnea is of great relevance for patient care and symptom management. Therefore, we recently developed and validated the Dyspnea-ALS-Scale (DALS-15) to detect and quantify the subjective sensation of dyspnea in a disease-specific manner [9]. The DALS-15 is a 15-item rating scale which is completed by the patients themselves, a so-called patient reported outcome (PRO). The use of a PRO instrument is advised when a concept may be better understood by the patient (e.g., severity of a symptom or state of a disease) [10]. The DALS-15 satisfies the rigorous criteria required by the Rasch model, allowing for quantification of dyspnea on an interval scale level [11]. One of the advantages of the DALS-15 is a high reliability index, the so-called Person Separation Index (PSI), with a value of 0.85 [9] indicating the ability of the scale to discriminate amongst respondents on an individual level. According to Tennant and Conaghan a minimum PSI of 0.7 is required for group-use and 0.85 for individual use of a scale [11]. Thus, the DALS-15 is applicable for individual patient evaluation and renders the assessment of dyspnea more standardized. It therefore can support wider acceptance of the patient reported experience of dyspnea as a criterion for NIV consideration in ALS.

Our recent study showed that the DALS-15 refers to dyspnea as a subjective component of respiratory impairment not captured by objective parameters like spirometric testing or blood gas analysis. In that study, up to 80% of patients with dyspnea according to the DALS-15 had spirometric test results and blood gas values still above the threshold values to initiate NIV (forced vital capacity (FVC) ≥50%, partial pressure of carbon dioxide (pCO_2_) < 45 mmHg) [12]. Nevertheless, in these patients NIV should be considered due to the presence of dyspnea. Patients and clinicians benefit from the DALS-15 in that it may help to improve the stratification of patients with respiratory impairment and to support decision-making in terms of indication for NIV. The DALS-15 may thus contribute to more efficient symptom management and coordination of care with major impact on quality of life in ALS.

We now focus on the diagnostic and clinical implications of the DALS-15 and the benefit for optimizing symptom management for individual patients in the daily clinical routine using a case-based approach.

## Methods

### Patients

This report presents two patients with dyspnea in ALS who were part of a larger study to develop and validate the DALS-15 [9]. They were exemplarily selected because they distinctiveley illustrate the diagnostic and clinical utility of the DALS-15 in clinical routine. Case 1 is representative for the group of patients in whom NIV should be considered although FVC and blood gas results do not reveal indication for NIV [12] and there is the striking finding of hypocapnia on presentation. The further clinical course of this case is valuable to demonstrate the clinical implications of the DALS-15 in later stages of respiratory impairment. Case 2 was considered representative for patients with advanced bulbar impairment in whom the DALS-15 can be of particular value due to loss of speech and the inability to perform spirometry.

The patients provided written informed consent before enrolment. They underwent pulmonary function tests and daytime blood gas analysis. Arterialized capillary blood was obtained from the earlobe at rest and analyzed using an ABL 800 Flex blood gas analyzer (Radiometer, Willich, Germany). Spirometry was performed with a portable Easy-One™ spirometer (ndd Medizintechnik AG, Zurich, Switzerland) using the software EasyWare 2012 Version 2.24.0.0. Measurements were recorded for the FVC in sitting and supine positions and were expressed as a percentage of the predicted value [13]. The percentage of decline in FVC upon changing from the upright to supine position was calculated (ΔFVC). A decline up to 10% can be considered normal and in case of ΔFVC > 25% in conjunction with restrictive pulmonary function relevant diaphragmatic weakness can be assumed [14].

Dyspnea was assessed by the DALS-15, which comprises 15 items [9]. It refers to the patient’s condition during the past 2 weeks using a 3-point Likert scale (0 = never, 1 = occasionally and 2 = often). The sum score ranges from 0 (no dyspnea) to a maximum of 30 points (severe dyspnea). The onset site of the first symptom was classified as bulbar or spinal (upper or lower limb, respiratory and axial) [15]. Physical functioning was assessed using the patient-rated ALS Functional Rating Scale - Extension (ALSFRS-EX) [16, 17] which consists of 15 items and is the extended version of the 12 items comprising ALSFRS - Revised (ALSFRS-R) [18]. As the latter scale is more commonly used, both scores are reported. Lower scores represent worse conditions.

## Results

### Case 1

A 72-year-old male patient with ALS suffered from moderate dyspnea (DALS-15 sum score: 14 out of 30 points) about 13 months after the diagnosis of ALS (spinal form with upper limb onset, ALSFRS-EX score 40/60, ALSFRS-R score 31/48). However, in tests of pulmonary function he still performed quite well (FVC upright 123%, FVC supine 104%, ΔFVC 15%). Blood gas results showed hypocapnia (pH 7.5, pCO_2_ 25 mmHg, partial pressure of oxygen (pO_2_) 89 mmHg, standard bicarbonate (sHCO_3_) 23 mmol/l, base excess (BE) -3.5). With regard to the results of the DALS-15 and after exclusion of other stress factors, the findings of the blood gas analysis were interpreted as hyperventilation (respiratory rate: 18 breaths per minute) due to the distressing sensation of dyspnea. The results of the spirometric tests and blood gas analysis did not point towards NIV. However, the DALS-15 identified dyspnea as an indicator for NIV consideration. As a consequence, the patient was referred to a sleep laboratory and nocturnal NIV was started. Six months later, the patient reported that dyspnea had increased in the preceding weeks, which was reflected by a higher DALS-15 sum score of 19 points. At the same time, we observed a rapid deterioration of the spirometric test results (FVC upright 74%, FVC supine 41%). The percentage of decline in FVC upon changing from the upright to supine position (ΔFVC) reached 45%, now indicating severe diaphragmatic weakness. The spirometric test results thus indicated NIV initiation 6 months later than the assessment of the subjective feeling of dyspnea by the DALS-15 did. Blood gas analysis now showed normocapnia. Due to the increase in the DALS-15 score and the rapid decline in the tests of pulmonary function, a scheduled appointment in the sleep laboratory was brought forward to optimize the ventilator settings. The patient was prescribed lorazepam 0.5 mg sublingually for episodes of paroxysmal breathlessness to reduce the associated anxiety and referral to a home palliative care service was recommended. Another 2 months later the patient was no longer able to visit our outpatient clinic and asked for a telephone consultation. He reported that dyspnea had even worsened and persisted at rest for some days now. He already received additional morphine. Nevertheless, dyspnea became a major burden (DALS-15 sum score: 24 points). He still declined tracheostomy and invasive ventilation. When asked about the current dosage of lorazepam, the patient said, that he had so far hesitated to take it. We addressed his concerns regarding lorazepam and strongly recommended to take this medication every four to 6 hours as needed. For further symptom relief we recommended to extend NIV into daytime hours beginning with about 3 h. Three weeks later the patient died peacefully at home.

### Case 2

Another 51-year-old male patient, who was diagnosed with bulbar ALS 13 months ago, attended the emergency department due to dyspnea and was admitted to the ward (ALSFRS-EX score 38/60, ALSFRS-R score 28/48). He was unable to communicate verbally and used a communication device. The severity and impact of dyspnea were evaluated using the DALS-15. This short bedside test with only ticking the answers did not bother the patient but gave us a good impression into the dyspnea-related distress the patient endured. According to the DALS-15, the patient suffered from severe dyspnea (DALS-15 sum score: 26 out of 30 points). He affirmed items of the DALS-15 representing a high symptom and emotional burden like “I have highly threatening dyspnea” (occasionally), “I am short of breath, while sitting still” (occasionally), “I wake up because of breathlessness at night” (often) and “I have fear of suffocation” (often). Blood gas analysis showed hypercapnia (pH 7.35, pCO_2_ 47 mmHg, pO_2_ 75 mmHg, sHCO_3_ 23 mmol/l, BE 4.6) corresponding to a later stage of respiratory impairment. Due to distinct bulbar impairment the patient could not perform spirometry. NIV was clearly indicated due to the blood gas analysis and the test results of the DALS-15 signaling the presence of severe dyspnea but was refused because of the patient’s claustrophobia. He decided against tracheostomy and invasive ventilation. Treatment of the patient’s respiratory complaints focused on secretion management and symptom-oriented drug administration beginning with morphine 2.5 mg orally three times daily for longer phases of dyspnea which occurred at rest and at night (see items of the DALS-15 above). Additionally, he was treated with lorazepam 0.5 mg two times a day sublingually and up to two rescue medications to reduce the affective distress caused by dyspnea (e.g. “fear of suffocation”). The patient was referred to a specialized palliative care team at his home place and died after 2 months.

## Discussion

Dyspnea clearly indicates a relevant impairment of respiratory function and is therefore the first stage of the recently proposed algorithm for initiation of NIV in patients with ALS [7]. Patient case 1 illustrates that the DALS-15 already detected dyspnea-related distress while FVC and blood gas analysis were quite well preserved. In this case the assessment of dyspnea through the DALS-15 thus could help to optimize coordination of care and symptom management. Initiation of NIV before onset of severe respiratory failure seems to improve adherence to the therapy [7]. Therefore, it is often helpful to give the patient time to get used to NIV, even though survival time is very limited when respiratory impairment is manifest in ALS. Patient 1 died within 9 months after the detection of dyspnea and there was a dramatic decline of the FVC of 50% during a period of 6 months. The rate of FVC decline and the onset of dyspnea are predictors of the individual ALS patient’s survival [6]. According to the literature, only 3% of the patients survive more than 2 years after onset of dyspnea [6]. According to Polkey et al. [19], the vital capacity declines slowly until 12 months before death, followed by a rapid decline until death as was also seen in our patient. The observation of hypocapnia in the first blood gas analysis in patient 1 was at the first glance a striking finding, because blood gas abnormalities in ALS rather relate to hypercapnia [20]. However, according to our recent study in a substantial study cohort of 70 patients with ALS suffering from dyspnea, 6% of the patients presented with hypocapnia [12]. Initial hyperventilation has also been described in a case series of patients with neuromuscular diseases who developed respiratory failure. Later during the disease course, as tidal volume decreases, alveolar ventilation is reduced first to normal and then to low levels resulting in hypercapnia [21].

Dyspnea despite well preserved parameters of pulmonary function in our patient on presentation suggests incipient respiratory failure. This supports the clinical relevance of the DALS-15 scoring in early stages of respiratory impairment. The assessment of dyspnea through the DALS-15 over a wide range of symptom severity is methodologically supported by the optimal targeting of the DALS-15 allowing for estimation of dyspnea with good accuracy from mild to severe dyspnea without a ceiling or floor effect [9]. Case 1 also illustrates that the DALS-15 is valuable for the guidance of patients in later stages of respiratory impairment when NIV is already implemented and dyspnea increases. Accordingly, the monitoring of dyspnea helped to optimize individual treatment in terms of determining the patient’s need for medication, the timing of NIV titration in the sleep laboratory and the adaptation of the duration of NIV sessions. A recent study showed that the positive effects of NIV on dyspnea do not carry over to periods of unassisted breathing. Consequently, there is a clear need for an increased awareness of the persistence of dyspnea-related distress during unassisted breathing [5]. In case 1 the DALS-15 showed that the patient still suffered from dyspnea-related distress despite NIV therapy indicating that the patient was therefore in a need for additional drug therapy for symptom relief. Also, in patient 2 the DALS-15 demonstrated the magnitude and implications of dyspnea for the patient’s daily living requiring urgent treatment whereas in this case drug therapy was the main approach. However, ALS-related dyspnea has so far received little attention beyond NIV and terminal care [5] and in contrast to other diseases there is only little evidence from the literature regarding dosage, efficacy and safety of drug therapy. So far only one study investigated morphine treatment in patients with terminal ALS more systematically, and demonstrated that therapeutic doses of morphine were safe and effectively reducing tachypnea and the intensity of dyspnea [22]. That lack of data may lead to uncertainty and tentativeness in treating ALS patients with dyspnea resulting in insufficient symptom management to the detriment of the patients. The application of the DALS-15 could be highly relevant as an outcome measure to capture the patients’ perspective in future clinical trials for pharmacological management of dyspnea.

In case 2, the DALS-15 was of benefit because the patient could indicate, non-verbally, how he was experiencing dyspnea. Despite this apparent impediment with loss of speech, the DALS-15 supported a standardized assessment of dyspnea-related distress and the diagnosis of respiratory impairment in a patient who was no longer able to perform spirometry due to severe bulbar impairment. In patients with bulbar or facial weakness many tests of pulmonary function cannot be adequately performed due to mouth leaks as they are unable to effectively seal their lips around the tube or mouthpiece. Moreover, vocal cord spasms, excessive saliva and gagging can interfere with test performance in these patients [23–26]. The DALS-15 allows for a standardized assessment independently of bulbar dysfunction. The development of the DALS-15 ensured that bulbar onset of the disease does not influence the probabilities of endorsing the items of the scale.

## Conclusions

Responding to dyspnea across the disease course is of high priority in patients with ALS and requires careful patient-centered assessment and quantification of dyspnea. This study shows the diagnostic and clinical implications and the benefit of the DALS-15 for individual patients in daily clinical routine. The use of the DALS-15 provides the opportunity to gain a deeper insight into the respiratory status of an individual patient and captures dyspnea-related distress as a subjective component of respiratory impairment in a standardized manner. It can help to detect dyspnea and diagnose respiratory impairment in patients in whom NIV should be considered due to the presence of dyspnea although FVC and blood gas values do not reveal indication for NIV. The implementation of the DALS-15 is also valuable for the guidance of patients in later stages when NIV is already introduced, and in patients with severe bulbar dysfunction in whom assessment of respiratory function is difficult due to loss of speech and inability to perform spirometric tests. The DALS-15 therefore has the potential to optimize individual treatment in ALS patients with dyspnea.

## Data Availability

All data generated or analysed during this study are included in this published article.

## Abbreviations

ALS: Amyotrophic lateral sclerosis
ALSFRS-EX: ALS Functional Rating Scale – Extension
BE: Base excess
DALS-15: Dyspnea-ALS-Scale
FVC: Forced vital capacity
NIV: Noninvasive ventilation
pCO_2_: Partial pressure of carbon dioxide
pO_2_: Partial pressure of oxygen
PRO: Patient reported outcome
PSI: Person separation index
sHCO_3_: Standard bicarbonate
ΔFVC: Percentage of decline in forced vital capacity upon changing from upright to supine position
